# Current State, Challenges, and Opportunities in Genome-Scale Resource Allocation Models: A Mathematical Perspective

**DOI:** 10.3390/metabo14070365

**Published:** 2024-06-28

**Authors:** Wheaton L. Schroeder, Patrick F. Suthers, Thomas C. Willis, Eric J. Mooney, Costas D. Maranas

**Affiliations:** 1 Department of Chemical Engineering, The Pennsylvania State University, University Park, PA 16802, USA; 2 The Center for Bioenergy Innovation, Oak Ridge, TN 37830, USA; 3 DOE Center for Advanced Bioenergy and Bioproducts Innovation, The Pennsylvania State University, University Park, PA 16802, USA; 4 Department of Biochemistry, Microbiology and Molecular Biology, The Pennsylvania State University, University Park, PA 16802, USA

**Keywords:** systems biology, computational biology, genome-scale modeling

## Abstract

Stoichiometric genome-scale metabolic models (generally abbreviated GSM, GSMM, or GEM) have had many applications in exploring phenotypes and guiding metabolic engineering interventions. Nevertheless, these models and predictions thereof can become limited as they do not directly account for protein cost, enzyme kinetics, and cell surface or volume proteome limitations. Lack of such mechanistic detail could lead to overly optimistic predictions and engineered strains. Initial efforts to correct these deficiencies were by the application of precursor tools for GSMs, such as flux balance analysis with molecular crowding. In the past decade, several frameworks have been introduced to incorporate proteome-related limitations using a genome-scale stoichiometric model as the reconstruction basis, which herein are called resource allocation models (RAMs). This review provides a broad overview of representative or commonly used existing RAM frameworks. This review discusses increasingly complex models, beginning with stoichiometric models to precursor to RAM frameworks to existing RAM frameworks. RAM frameworks are broadly divided into two categories: coarse-grained and fine-grained, with different strengths and challenges. Discussion includes pinpointing their utility, data needs, highlighting framework strengths and limitations, and appropriateness to various research endeavors, largely through contrasting their mathematical frameworks. Finally, promising future applications of RAMs are discussed.

## 1. Introduction

Metabolic modeling (i.e., mathematically and computationally representing the biochemical processes occurring in the context of an organism’s metabolism) is an important and increasingly used tool in systems biology. Early metabolic models were stoichiometric models of metabolism (SMMs). Such models empower mathematical and systematic frameworks (i.e., paradigms of methodology and workflows) for integrating and evaluating large-scale reaction networks [1] and are enabling of numerous potential applications. Perhaps the most widespread application is the in silico design of microbial cellular factories [2,3], with analysis and design thereof accomplished using established tools for network manipulations and analysis [2,4]. Other applications range from (re)evaluation of in vivo data to elucidate mechanisms and phenomena which are expensive or difficult to measure or are otherwise unmeasured [5,6,7] to drug target identification and development [7,8,9]. Many such applications of metabolic modeling using SMMs have been recently reviewed [9,10].

Despite their extensive successful applications, SMMs are limited in their predictive capability as they do not explicitly track the costs of protein beyond its bulk contribution to biomass. Furthermore, they do not directly incorporate mechanistic detail that can reduce the solution space such as enzyme kinetic capacity or physical proteome limitations, crowding, degradation, and dilution through growth and cell division. Having models that can more accurately account for these items could safeguard against overly optimistic phenotype predictions. At present, there are several types of modeling frameworks, each with its own strengths, areas of applicability, and data requirements. Here, we will discuss those which go beyond simple stoichiometric genome-scale models of metabolism and integrate the cost to metabolism for enzymatic catalysis in the mathematical problems which form the model. These models are reconstructed through one of several current protein constraint frameworks which integrate enzyme capacity, cost, and simple kinetic limitations at genome scale. The mathematical form of these problems ranges in solution complexity. Some are relatively straightforward linear programming (LP) problems that only contain continuous variables with each equation expression a polynomial of degree one or less. In some cases, LP is applied iteratively to reduce computational complexity by avoiding non-linear programming (NLP). NLP is also sometimes used in place of iterative LP. Finally, more computationally demanding mixed-integer linear programming (MILP) problems that are also linear but contain some variables that can only take on integer values.

As the field of metabolic modeling has evolved, it has resulted in instances of unclear or inconsistent terminology, which should briefly be discussed so that all readers will have the same understanding of the terms used herein. As previously stated, a model is the mathematical representation of the biochemical processes occurring in an organism’s metabolism. A framework is the methodology and/or workflow by which a model is created which often determines the form of the mathematical representation. The term scale will be used to describe the size or breadth of a model. From these fairly basic definitions, inconsistencies in the meaning of terms arise quickly. One of the most commonly used terms, genome-scale (GS) is one such term. Conventionally, the term GS has been synonymous with SMM models [1,11], indicating that the stoichiometric metabolic model accounts for all metabolic functions supported by its current gene sequence, genome annotation, and available biochemical data. These models were often referred to as GSM, GSMM, GEM, or M-models. However, the term GS has recently also been applied to describe the scale of other model types. For example, it has been used to describe a resource allocation model (RAM) [12]. Also, there has been considerable attention as to how to achieve GS kinetic models of metabolism [13,14], with some kinetic models already described as “near genome-scale” [15]. Given its expanding definition, here we will follow the newer convention of using the GS descriptor to indicate model scale, not type. Models which are purely stoichiometric, such as *i*CTH669 [5], will be referred to as stoichiometric metabolic models (SMMs) to avoid confusing model type and scale.

Similarly, we note that significant disagreement in terminology exists for models which account for protein or enzyme synthesis and capacity in metabolic modeling (i.e., the focus of this review). We address the differing terminologies used throughout this field briefly to familiarize the reader with differing descriptions of the same idea, as well as to establish the definition of convenient terms used throughout this review. Some works have referred to such models as resource balance analysis (RBA) models (example: scRBA) [16]; other authors have referred to such models as resource allocation models (RAMs) [12,17,18], others as proteome- or enzyme-constrained genome-scale models (ecGEMs or pcGEMs) [19], or ME-models (where “ME” stands for metabolism and macromolecular expression) [20,21,22,23]. For convenience, we will follow the convention used by two recent reviews [17,24] describing all models which account for protein or enzyme synthesis and capacity in models of metabolism under the umbrella term of resource allocation models (RAMs). We reserve the use of the terms RBA and ME-models to specific realizations of models constructed using their respective modeling framework, such as the scRBA model [16].

Herein, we provide a broad discussion of precursor tools for accounting for protein cost (such as flux balance analysis with molecular crowding (FBAwMC)) and current RAM frameworks and some of their applications. Specifically, the first part pinpoints their utility, data needs, and appropriateness to various research endeavors, through examining and contrasting their mathematical frameworks. The second part details challenges and opportunities these frameworks can help address or can be expanded to address. The third part addresses the current divergent landscape of frameworks and vocabulary and advocates for community standards regarding language and naming conventions. Finally, we draw conclusions and posit promising future directions for RAMs in medicine, bioproduction, and agriculture.

## 2. Stoichiometric Models of Metabolism (SMMs) and Flux Balance Analysis (FBA)

Stoichiometric models of metabolism (SMMs) are the forerunners of RAMs and are often wholly or largely incorporated into GS RAMs [16,20]. They therefore form an appropriate starting point for the discussion of RAMs. SMMs are mathematical representations of the network of metabolic reactions in an organism and are used to explore possible phenotypes of said organism, typically during exponential growth phase. They contain a comprehensive list of both metabolites and reactions occurring within the organism and environment, organized as a stoichiometric matrix, as well as a set of bounds for the flux of each reaction. The stoichiometric matrix (detailing the stoichiometry of each modeled reaction), flux bounds (which specify reaction direction and reversibility), gene-protein-reaction associations (GPR), and ATP maintenance values form the core of SMMs [11,25]. In building the stoichiometric matrix, gene-protein-reaction (GPR) relationships linking the genome to metabolism are established. A biomass description in the form of a biomass pseudo-reaction is added to the stoichiometric matrix for modeling growth. Finally, maintenance costs, in growth and non-growth associated maintenance (GAM and NGAM, respectively), define an ATP-based cost to non-modeled cellular functions [11,25]. The most basic analysis of SMMs is called flux balance analysis (FBA) which seeks to maximize or minimize a particular objective function (e.g., biomass yield, product yield, substrate uptake, etc.) subject to mass balance at pseudo-steady state [25]. Mathematically, this objective is represented as:

(1) $$min\;or\;max\;z=c_{j}v_{j}$$

where z is the objective variable, J is the set of reactions, $c_{j}$ is a vector of the objective weight for reaction j, and $v_{j}$ is the flux through reaction j (in mmol gDW^−1^ h^−1^). FBA is a linear programming (LP) problem formulated so that it seeks to solve Objective (1) as follows:

Subject to (s.t.)

(2) $$\sum_{j\in J}S_{ij}v_{j}=0\;\;\;\;\;\;\;\;\;\;\;\;\;\;\;\;\;\;\;\;\;\;\forall j\in J$$

(3) $$v_{j}^{LB}\leq v_{j}\leq v_{j}^{UB}\;\;\;\;\;\;\;\;\;\;\;\;\;\;\;\;\forall j\in J$$

where I is the set of metabolites, $S_{ij}$ is the stoichiometric matrix expressing participation of metabolites in corresponding reactions with the sign denoting reactants (negative) or products (positive), $v_{j}^{LB}$ are the lower bounds for $v_{j}$, and $v_{j}^{UB}$ are the upper bounds for $v_{j}$. Constraint (2) is the steady-state assumption, assuming that metabolite concentration is unchanging at the time scale of *FBA* (e.g., a “snapshot” of metabolism). Different frameworks use different objective weight vectors, but typically, $c_{j}=0$ for all reactions other than the reaction corresponding to the desired biological optimization goal.

**Figure 1:**
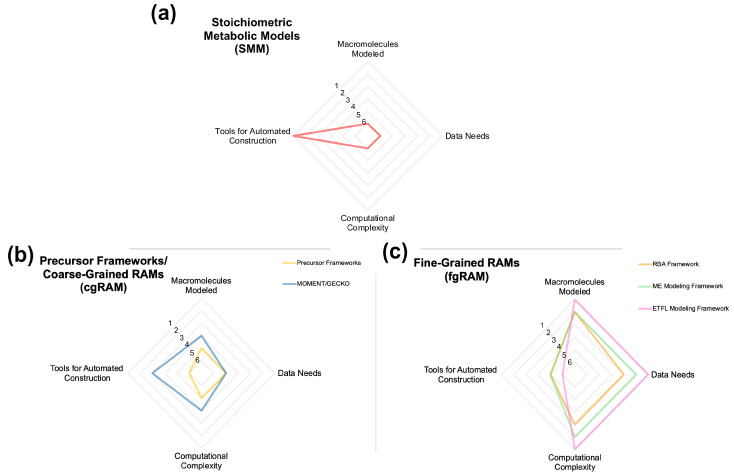
Radar graphs depicting the compared abilities and constraints of a given model type for (**a**) stoichiometric metabolic models, (**b**) precursor frameworks and coarse-grained resource allocation models (cgRAM), and (**c**) fine-grained resource allocation models (fgRAM). Each of the four categories is assigned an arbitrary value 1–6, designating a comparative “ranking” between the six modeling types within each category (e.g., a value of “1” designates the highest amount within a category, “2” designates second highest, and so on). When necessary, equivalent values are assigned to model types that have no meaningful distinction within a given category. Descriptions of MOMENT and GECKO frameworks can be found in Section 4.1.1 and Section 4.1.2, respectively. Descriptions of RBA, ME Modeling, and ETFL modeling frameworks can be found in Section 4.2.1, Section 4.2.2 and Section 4.2.3, respectively.

As a result of their form and computation, stoichiometric GSMs are often reconstructed soon after a modeled organism is sequenced as data needs for a first draft model are minimal. For example, the first sequence of *Escherichia coli* was published in 1997 [26], with its first stoichiometric GSM following only three years later [27]. Since then, the number of SMMs has increased dramatically, reaching 6239 organisms by 2019 [10]—albeit with a vast range in the extent of curation. Some larger recent efforts, such as AGORA2, have exceeded that mark, generating 7302 draft stoichiometric GSMs in a single study [28]. Figure 1a summarizes the features of SMM that are relevant to this review. Curated SMMs have been rather successful in addressing a wide range of research questions, testing hypotheses, and generating testable hypotheses [10,29].

**Figure 2:**
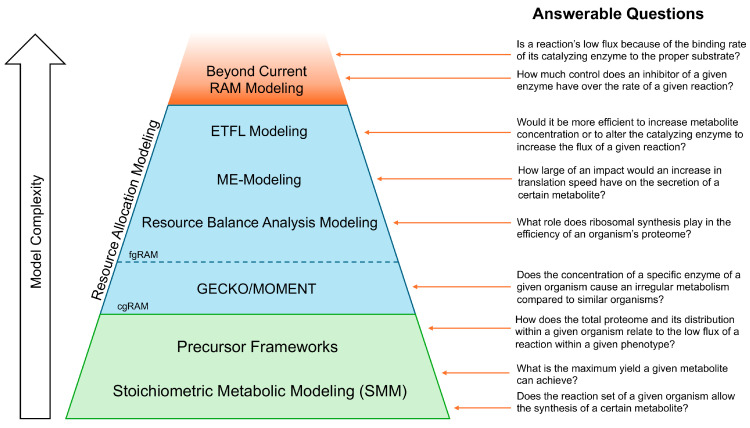
Pictorial representation of example research questions that can be answered with various model types. The trapezoidal shape showcases the relative number of model types that can answer a given question, with all models above a given question able to provide an answer, and all models below unable to do so.

However, being purely stoichiometric representations reconstructed from genomic and direct biochemical data [11], these models face several inherent limitations. First, in vivo reaction fluxes are constrained by thermodynamics, the abundance of enzymes, and enzyme kinetics, among other factors. However, within SMMs, aside from specifying directionality, allowable bounds are set to arbitrarily large values which can be too permissive. Many cellular phenotypes (i.e., growth rates, flux distribution, or product yield) remain stoichiometrically feasible but are physiologically unrealizable. Second, SMMs do not capture quantitatively genome to proteome to metabolome relations beyond simple mapping information (i.e., GPRs). The cost of macromolecular synthesis and maintenance is captured in an aggregated manner within simple metrics such as growth-associated maintenance (i.e., GAM) or the non-growth associated equivalent, NGAM. Third, whereas the biomass description contains provisions for accounting for amino acid needs for protein synthesis in aggregate, no specific connection is made between the amount of enzyme synthesized and the possible flux of the associated reaction(s). Finally, physical limitations by cellular and organelle size and area impose additional limits to their total proteome (i.e., volume of cytosol, membrane surface area, etc.). In other words, the machinery of metabolism is limited by the products of metabolism. Interesting questions arising from the limitations of protein allocation are illustrated with Figure 2.

## 3. Precursor Frameworks

Flux profiles determined by optimization for SMMs sometimes tend towards extremal states that may be physiologically unreachable. To address these shortcomings, resource allocation models (RAMs) were developed, building upon SMMs with additional constraints on metabolic flux, macromolecular synthesis networks, and/or information flow (i.e., transcription and translation rates). Prior to the development of full RAMs, there were a few ad hoc SMM tools which sought to address these limitations. One of the first such attempts was the Flux Balance Analysis with Molecular Crowding (FBAwMC) [30], which limited the total volume of metabolic enzymes. Another effort, FBA with solvent capacity constraints, followed similar mathematical principles, yet was based on total enzyme concentration [31]. This latter framework has been particularly influential in RAM modeling frameworks. Another framework [32] applied weighting coefficients based on enzyme burden to direct metabolic flux on a pathway-scope (e.g., glycolysis and pentose phosphate pathways) down optimal paths. These efforts, however, are mathematical tools applied to SMMs, rather than new modeling frameworks.

**Figure 3:**
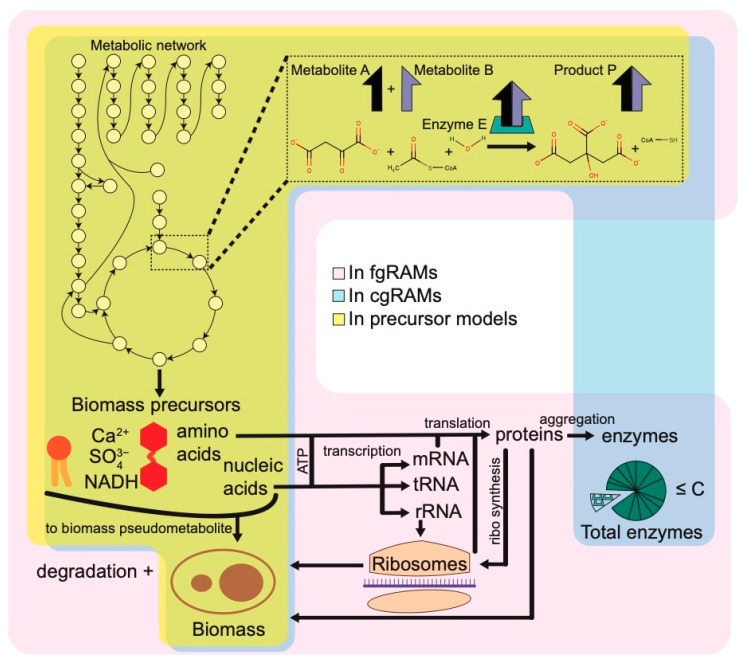
Diagram of components in precursor, cgRAM, and fgRAM models. For each model group, any element included in at least one model (e.g., in RBA but no other fgRAMs) is included. Areas included in each model group are translucent, so aspects found in multiple are noted by overlapping colors.

Since these early efforts, several different modeling frameworks or analysis tools for SMM models which constrain metabolism through allocation of protein resource have been developed. In general, these frameworks limit either the total abundance of protein or of individual proteins, but they lack explicit per-reaction constraints limiting flux in accordance with a kinetic parameter. Here, we briefly discuss two influential tools. All precursor SMM tools are linear programming problems (LPs). Their requirements and their abilities are summarized in Figure 1b, and the modeling framework is summarized in Figure 3.

### 3.1. Flux Balance Analysis with Molecular Crowding (FBAwMC)

Flux balance analysis with molecular crowding (FBAwMC) [30] sought to apply the limitations of cellular volume to FBA. It uses Constraints (1) to (3) as well as a new cellular volume limitation constraint as shown below:

(4) $$\sum_{j\in J}\frac{\rho_{j}C}{k_{val}}v_{i}=\sum_{j\in J}a_{j}v_{i}\leq 1\;\;\;\;\;\;\;\;\;\;\;\;\;\;\;\;\;\;\;\;\;\;\;\;\;\;\;\;\forall j\in J$$

where $\rho_{j}$ is the volume (mL) per mmol enzyme associated with reaction j, $k_{val}$ is a kinetic parameter (not necessarily $k_{cat}$, h^−1^), and C is the cytoplasmic density, in g mL^−1^. Taken together, these terms define the parameter $a_{j}$ which is defined as the crowding coefficient of reaction j (gDW h^−1^ mmol^−1^). FBAwMC has been used to predict metabolic switching between high and low yield pathways and its relation to redox metabolism [33], predict growth rate and substrate utilization of mutant *E. coli* strains [30], and identify regulatory mechanisms controlling metabolic switches between states [34]. Given the nature of the new bounding constraint introduced, FBAwMC can be run without needing additional data beyond a typical SMM. However, to elucidate meaningful information on the kinetics of various fluxes, enzyme volume and cytoplasmic density data are necessary (see Figure 1b for a comparison of data needs between different modeling frameworks).

### 3.2. FBA with Solvent Capacity Constraints (FBAwSCC)

Solvent capacity constraints, as in FBAwMC [30], limit the total amount of proteome in the cell as a fraction of the total biomass in FBA with solvent capacity constraints (FBAwSCC) [31]. For this framework, the enzyme capacity constraint is as follows:

(5) $$\sum_{j\in J}\frac{MW_{j}v_{j}}{k_{cat,j}}\leq C\;\;\;\;\;\;\;\;\;\;\;\;\;\;\;\;\;\;\;\;\;\;\;\forall j\in J$$

where $MW_{j}$ is the molecular weight of the protein associated with reaction j (in g mmol^−1^), $k_{cat,j}$ is the enzyme turnover number (in h^−1^) of the enzyme associated with reaction j, and C is the limit on metabolic enzyme concentration in g gDW^−1^. This constraint was used to model the Warburg effect in proliferating cancer cells, which is a metabolic phenotype with high glycolytic flux and lactic acid fermentation [31]. Outside the typical SMM data needs, kinetic and molecular weight data are necessary to introduce the new constraint, although much of these data can be found in online databases, such as BRENDA [35]. However, an accurate limit on the metabolic enzyme concentration may need to be acquired from explicitly performed proteomics experiments (see Figure 1b for a comparison of data needs between different modeling frameworks). To our knowledge, FBAwSCC has only been applied once; however, this framework is influential in that some of the RAM frameworks utilize a very similar solute capacity constraint.

## 4. Resource Allocation Model (RAM) Frameworks

Broadly, RAM frameworks fall into two categories: those which do not impose metabolic costs for the synthesis of macromolecules, and those which do. Models which do not require macromolecule synthesis have been referred to as enzyme-constrained GEM (ecGEM) [24,36] and coarse-grained pcGEM [37] models. Herein, the term coarse-grained RAM (cgRAM) will be used to describe these models. The latter category, which explicitly imposes metabolic costs, includes RBA models, ME-models, and ETFL models. These model types are more complex, leading to a non-static biomass composition, though generally at the cost of requiring more data. These models have confusingly also been called proteome-constrained GEM [36], enzyme-constrained GEMs (ecGEM) [24], RAM [24], and fine-grained proteome-constrained (pcGEM) [38] models. For this review, since the term RAM is used inclusively for all protein-constrained metabolic models, the term fine-grained RAM (fgRAM) will be used. A visual summary of various RAM frameworks is given in Figure 3, highlighting the different constraints and what is explicitly modeled in the flow of metabolites and biological information within a cell.

### 4.1. Coarse-Grained RAMs (cgRAMs)

Coarse-grained RAMs are easier to reconstruct and analyze because only some of the steps of the central dogma of biology are explicitly modeled. Therefore, information on enzyme degradation rates, ribosome efficiency, and global proteomic measurements are not needed. These frameworks are based on the structure of stoichiometric models, but with two new key constraints added.

The first constraint limits reaction fluxes in proportion to the enzyme concentration and a single kinetic parameter in the form

(6) $$v_{j}\leq k_{val}e_{j}\;\;\;\;\;\;\;\;\;\;\;\;\;\;\;\;\;\;\;\;\forall j\in J$$

where $k_{val}$ is a kinetic parameter (in h^−1^, often $k_{cat}$ for cgRAMs [39,40,41]), and $e_{j}$ is the concentration of enzyme e catalyzing reaction j in mmol gDW^−1^. Generally, $k_{cat}$ values are extracted from a database such as BRENDA [35], though conceivably an apparent kinetic parameter ($k_{app}$) could be used to correct for kinetics overestimation where substrate saturation is low. Depending on the framework, this constraint is modified to account for scenarios where an enzyme complex catalyzes a reaction (“and” GPR logic, Constraint (7) below) or where multiple isozymes catalyze a single reaction (“or” GPR logic, Constraint (8) below):

(7) $$v_{j}=k_{val}\min\left(e_{j,1},e_{j,2},\ldots ,e_{j,n}\right)\;\;\;\;\;\;\;\;\;\;\;\;\;\;\;\;\;\;\;\;\;\;\forall j\in J$$

(8) $$v_{j}=k_{val}\sum_{e_{j}\in E^{j}}e_{j}\;\;\;\;\;\;\;\;\;\;\;\;\;\;\;\;\;\;\;\;\;\;\;\;\;\;\;\;\;\;\;\;\;\;\;\;\;\;\;\;\;\;\;\;\;\;\;\;\forall j\in J$$

where $E^{j}$ is the set of isozymes capable of catalyzing reaction j.

The second key constraint in cgRAM models is a limit on the total protein or enzyme concentration in the cell. This constraint takes various specific forms, following the general form below, similar to Constraints (4) and (5) of the precursor frameworks FBAwMC and FBAwSCC, respectively:

(9) $$\sum_{e\in E}e_{e}MW_{e}\leq C$$

where E is the set of all modeled enzymes, $MW_{e}$ is the molecular weight of enzyme e, and C is some upper limit on total enzyme concentration (making it more analogous to FBAwSCC than FBAwMC). The exact formulation of this constraint varies between frameworks. Constraint (9) renders protein allocation at high levels of metabolic activity into a zero-sum game, implying that more enzyme for one pathway means less enzyme capacity for others. C should therefore be interpreted as the overall enzyme concentration limit and be carefully chosen. The result of these models are flux distributions and enzyme concentrations.

Only a limited amount of data is required for cgRAM parameterization beyond what is already included in SMMs (i.e., reaction stoichiometry, directionality, gene-protein-reaction links) as reflected in Figure 1b. At minimum, cgRAM reconstruction also requires $k_{cat}$ value estimates, molecular weights from each enzyme, and an estimate for C present in Equation (9). Approximate $k_{cat}$ values and protein sequence data can be obtained from databases such as BRENDA [35], SABIO-RK [42], and UniProt [43]. Of course, database $k_{cat}$ values refer to in vitro measurements which may differ considerably from in vivo values. Thus, cgRAM models can be reconstructed using only database-derived information. In general, cgRAM models are dominated by two frameworks: MOMENT and GECKO (and their progeny or variants thereof). Their requirements and capabilities are summarized graphically in Figure 2b, and the constituents of a cgRAM model are shown in Figure 3.

#### 4.1.1. Metabolic Modeling with Enzyme Kinetics (MOMENT) Framework and Successors

The MOMENT framework [41] utilizes Constraints (1) through (3) and (6) through (9). It was first demonstrated by applying it to the *i*AF1260 model of *E. coli.* In Constraint (9), C represents the total weight of proteins in g gDW^−1^ in the cell. A follow up of MOMENT, referred to as short MOMENT (sMOMENT), reformulates Constraints (6) and (9) to reduce the number of model constraints as shown below [44]:

(10) $$-\left(\sum_{j\in J}v_{j}\frac{MW_{j}}{k_{cat,j}}\right)+M_{Pool}=0$$

(11) $$M_{Pool}\leq P_{tot}$$

where $P_{tot}$ is the total protein concentration, $M_{Pool}$ is the mass of all metabolic enzymes needed to catalyze all reaction fluxes, and $MW_{j}$ is the molecular weight of the enzyme associated with reaction j. Essentially, this reformulation removes variables $e_{j}$. Note that Constraints (10) and (11) taken together is equivalent to Constraint (5) of FBAwSCC except that C is replaced with $P_{tot}$ and its enforcement as an upper bound is now handled through the intermediary $M_{Pool}$ variable. This alteration somewhat blurs the line between what does and does not constitute an RAM. We refer to sMOMENT as an RAM as it identifies as a modeling framework, whereas FBAwMC and FBAwSCC identify as FBA tools for SMMs and so we classify them as such. Notably, sMOMENT is introduced alongside AutoPACMEN, an automated workflow for generating sMOMENT models given a Systems Biology Markup Language (SBML) [45] formatted SMM [44]. MOMENT and sMOMENT models are generally formatted as SBML models, with AutoPACMEN implemented as a module in the Python package COBRApy [46]. The AutoPACMEN framework is available through GitHub (https://github.com/klamt-lab/autopacmen, accessed on 24 June 2024).

MOMENT models have been used to model the change in fermentation product yield in *E. coli* with increased glucose uptake [44], evaluating predicted growth rates of *E. coli* on various carbon sources [41], and evaluating proteome distribution efficiency in *E. coli* [47].

As with all cgRAM models, MOMENT models are relatively easy to reconstruct, and require datasets from well-established databases. This feature makes MOMENT models more accessible for under-studied organisms. MOMENT models are also less computationally complex than models reconstructed in other RAM, particularly using sMOMENT. MOMENT models are also relatively consistent in formatting using the systems biology markup language (SBML), which is commonly used with SMMs. However, there is no explicit metabolic cost for the proteome, and total protein distribution is limited by a single constant (C of $P_{tot}$). A poor selection of this constant may render a MOMENT reconstruction with similar behavior to an SMM (where the constant is too large) or with unrealistically sluggish metabolism (where the constant is too small). A relatively simple future development to partially overcome this problem could be the definition of a “dummy” protein, the synthesis rate for which replaces C of $P_{tot}$. Doing so would limit cell total protein content while creating a metabolic burden with relatively minimal increase in computational complexity (i.e., the introduction of a single reaction).

#### 4.1.2. GEM with Enzymatic Constraints Using Kinetics and Omics (GECKO) Framework and Its Progeny

The GECKO framework was first introduced in 2017 [39] by expanding upon the Yeast7 SMM of *Saccharomyces cerevisiae*. In analogy to MOMENT, GECKO is defined by constraints (1) through (3), (6), and (9). GECKO, however, incorporates enzymes directly into the stoichiometric matrix. A reaction catalyzed by enzyme $e_{j}$, $e_{j}$ is treated as an additional substrate with a coefficient of $k_{cat}^{-1}$. In Constraint (9), parameter C accounts for protein saturation and the fraction of metabolic enzymes, as shown below:

(12) $$C=\sigma fP_{tot}$$

where σ is the protein saturation (i.e., what fraction of the maximum protein concentration is currently being used) and f is the fraction of enzymes accounted for in the model (i.e., metabolic enzymes). GECKO 2.0 expands on the GECKO framework by integrating within the COBRA toolbox for MATLAB [38] and the Python package COBRApy [46]. Additional improvements include improved automated $k_{cat}$ gathering, new utilities, and more flexible input formats [46]. More recently, the protein allocation adjustment for alternative environments (PARROT) framework [40] builds upon GECKO 2.0. PARROT minimizes the distance between enzyme allocation in a reference state, $E_{ref}$, compared to an alternative growth condition, $E_{s}$, using weighted or unweighted Manhattan or Euclidean distances. Model file types are in plain text and SBML formats. Models constructed by following GECKO and PARROT frameworks both generate proteome allocation predictions. GECKO is available through GitHub (https://github.com/SysBioChalmers/GECKO, accessed on 24 June 2024), which as of the time of writing has advanced to GECKO 3.0. GECKO 3.0 is published as a protocol for model reconstruction which integrates deep learning-predicted enzyme kinetics [48]. GECKO models have been used to design a biosynthetic pathway for poly-γ-glutamic acid in *B. subtilis* [49], studying long-term adaptation to stress through proteomics incorporation in budding yeasts [50], and to explore anoxic metabolism and metabolic cooperation in tumor cell microenvironments [51].

The GECKO framework has relatively similar strengths, weaknesses, and potential for future development to the MOMENT framework. However, the strong programming support for GECKO, with continual improvement up to version 3.0 at the time of writing, and implementation as a package in MATLAB and python, in addition to the recently published protocol and integration of deep-learning predicted enzyme kinetics [48], provide a substantial implementation and support advantage in this framework compared to MOMENT.

#### 4.1.3. Automated Reconstruction of MOMENT and GECKO Models

The reconstruction of cgRAM models has largely been automated. The first tool, codeveloped with sMOMENT, but also capable of generating GECKO models, was the automatic integration of protein allocation constraints in metabolic networks (AutoPACMEN) tool [44]. It uses SABIO-RK, BRENDA, and optional user-provided databases for assigning $k_{cat}$ values. For cases with unknown $k_{cat}$ values, median or mean values are used. This effort was shortly followed by the Python-based workflow for constructing enzymatic constrained metabolic network models (ECMpy), first demonstrated using the *i*ML1515 SMM of *E. coli*. ECMpy has a slightly different formalism on the total enzyme constraint,

(13) $$\sum_{j\in J}\frac{v_{j}MW_{j}}{\sigma_{i}k_{cat,j}}\leq P_{tot}f$$

where $\sigma_{i}$ is enzyme saturation (default value of 1). This single constraint is a condensation of Constraints (9) through (12), making the framework compatible with both MOMENT and GECKO. Specifically, for a MOMENT model $f,\sigma_{i}=1$ and $C=P_{tot}$, whereas for a GECKO model $\sigma_{i}=1$ and $e_{j}=v_{j}/k_{cat,j}$ as defined in the GECKO mass balance. ECMpy has a python-based cgRAM reconstruction workflow available through GitHub (https://github.com/tibbdc/ECMpy, accessed on 24 June 2024), and is now on its second version [52] Notably, successive iterations of GECKO reconstructive workflows have been created, the most recent being GECKO 3.0 [48], and are available through GitHub (https://github.com/SysBioChalmers/GECKO, accessed on 24 June 2024).

### 4.2. Fine-Grained RAMs (fgRAMs)

As with cgRAMs, fgRAMs contain the basic FBA framework constraints (constraints (1) to (3)), and basic cgRAM constraints (Constraints (6) and (9)). fgRAMs expand upon cgRAMs by requiring the synthesis of macromolecules, particularly metabolic enzymes from products of metabolism (e.g., amino acids, ATP, GTP) which in turn limits their production (e.g., enzyme catalytic activity). As these models are at pseudo-steady state and do not include metabolite concentrations, synthesis rates are instead used to impose metabolic burden for macromolecules, as well as to bound their activities. The use of synthesis rate is based on the steady-state assumption (i.e., that macromolecular concentrations do not change). These frameworks generally use three drains on macromolecular concentration which their synthesis must match: degradation, dilution, and consumption, which the synthesis rate must match. This constraint takes the following form.

(14) $$v_{m}=k_{deg,m}e_{m}+\mu e_{m}+v_{con,m}\;\;\;\;\;\;\;\;\;\;\;\;\;\;\;\;\;\;\;\forall m\in M$$

where M is the set of macromolecules modeled (note E⊂M), $v_{m}$ is the flux through the macromolecule synthesis reaction (in that macromolecular synthesis is incorporated into the stoichiometric matrix), $k_{deg,m}$ is a degradation constant for macromolecule m, $e_{m}$ is the concentration of the macromolecule m, μ is the growth rate of the organism (representing the loss in concentration occurring from dilution), and $v_{con,m}$ is the rate of macromolecule m consumption. From the rate of enzyme synthesis, Constraint (6) is modified slightly to the following:

(15) $$v_{j}\leq \frac{k_{app}v_{e,j}}{\mu }\;\;\;\;\;\;\;\;\;\;\;\;\;\;\;\;\;\;\;\;\;\;\;\;\;\forall j\in J,\;e\in E$$

where $v_{e,j}$ is the flux through the enzyme synthesis reaction for enzyme e catalyzing reaction j and $k_{app}$ is the apparent kinetic parameter. Note that in many frameworks, such as Resource Balance Analysis (RBA) modeling, Constraint (15) is used to tune or calculate $k_{app}$ values based on proteomic and fluxomic datasets (such that metabolic flux and enzyme abundance can change for different growth conditions).

A notable feature of fgRAM models distinguishing them from cgRAM models is their semi-variable biomass composition. The flux through the metabolic network is dependent on macromolecular synthesis rate, and these macromolecules constitute the majority of biomass. Therefore, the weight of biomass components synthesized are tracked and weight ratios enforced to produce a biomass reaction or pseudo-metabolite of appropriate weight. Therefore, the composition of protein, RNA, DNA, or other macromolecular components of biomass is dependent on the metabolic state. Their requirements prediction range are summarized graphically in Figure 1c, and the components of cgRAM framework are summarized in Figure 3.

Generally, fgRAMs are solved by either an iterative LP or mixed-integer linear programming (MILP) approach, increasing the computational complexity of the models compared to cgRAM and precursor frameworks. Below, we discuss three representative fgRAM frameworks. Although this list is not exhaustive, we believe that these three highlight representative trends within fgRAM modeling and at present are the most influential and widely used frameworks in this space. Other frameworks include the resource constrained FBA framework [53] and the deFBA framework [54].

#### 4.2.1. Resource Balance Analysis (RBA)

The first RBA framework was developed in 2011 to model *Bacillus subtilis*, using a previously published but unnamed SMM as a basis for the metabolic network [55] and was expanded upon in 2015 [56]. More recently, updates and extensions to this framework were inspired from the deFBA framework by Reimers et al. [18]. These extensions include organelles and separate tracking of mitochondrial proteins, and was applied to RAM reconstruction scRBA, which expanded upon the SMM *i*Sace1144 [16]. This extended framework will be used as the mathematical description of the RBA framework, as it has a comprehensive formulation and straightforward workflow. Through utilizing Equation (16), RBA frameworks assume $k_{deg,m},v_{con}=0$ for all macromolecules, and thus the synthesis rate of macromolecules is balanced only by dilution. Furthermore, whereas macromolecules such as RNA, DNA, lipids, and proteins among others constitute biomass, RBA models only consider the cost of protein and RNA synthesis. This assumption is generally reasonable as these are the two largest fractions of cell dry weight (55% and 20%, respectively in *E. coli* [57]). As such, biomass is generally variable in amino acid and RNA composition and fixed in terms of other biomass contributors (though the scRBA model does add biomass variability because of growth rate [16]). Given that enzyme synthesis, not concentration, is used, Constraint (9) is implemented in RBA models with the following modification:

(16) $$\frac{1}{\mu }\sum_{p\in P}v_{p}MW_{p}\leq C$$

where P is the set of proteins, $v_{p}$ is the rate of protein synthesis, and $MW_{p}$ is the protein molecular weight. Note that depending on the framework, these equations may be arranged differently, but we will present framework constraints as close to forms already presented as possible to highlight similarities and/or differences. Constraint (16) is functionally equivalent to Constraint (9) on a protein basis (rather than an enzyme basis) noting that $v_{p}/\mu =e_{p}$ in the case of no degradation or consumption. This same equality also means Constraint (15) is equivalent to Constraint (6) in an RBA model. RBA models use two additional and new constraints. First, a limit on rRNA capacity is imposed:

(17) $$\frac{1}{\mu }\sum_{r\in R^{r}}v_{r}MW_{r}\leq C_{r}$$

where $R^{r}$ is the set of rRNA molecules in the organism, $MW_{r}$ is the molecular weight of r, and $C_{r}$ is an upper bound (in g gDW^−1^) on rRNA abundance (the same base symbol denotes its identical role to C, except applied to rRNA). Second, protein synthesis is limited by the capacity of ribosomes in the protein-ribosome coupling constraint, shown below:

(18) $$k_{ribo}v_{ribo}\leq \mu \sum_{p\in P}N_{p}^{aa}v_{p}$$

where $k_{ribo}$ is the rate at which ribosomes elongate peptides (in amino acids s^−1^) and $N_{p}^{aa}$ is the number of amino acids in protein p. In total then, RBA models use Constraints (1) through (3), and (15) through (18).

Another consideration in the RBA framework is that not all synthesized proteins have a metabolic role. Therefore, these frameworks generally enforce some maximum fraction of the total protein sum that is allowed to be metabolic protein, with the remainder regrettably referred to as “dummy” protein. Beyond the data inherent in cgRAM frameworks, RBA models require the definition of a dummy protein, $k_{app}$ values (or proteomics and fluxomics from which to calculate them), an estimate of the fraction of proteome that is metabolic, and detailed knowledge about the aggregation of proteins into enzyme (e.g., heteromers, homomers).

RBA models are analyzed using Resource Balance Analysis, the analysis technique inherent in the modeling framework. In the analyses of these models, iterative linear programming is used. The iterative steps are used to maximize growth rate, and the objective function used is generally to minimize protein synthesis [16]. The results of these analyses are a single metabolic state, akin to flux balance analysis (FBA).

For manual reconstruction of RBA models a protocol [58] and a workflow [16] have been developed. There are some tools for automating RBA model reconstruction and analysis. A recent Python package, RBApy [59], has been created to automatically reconstruct RBA models from genome annotations in XML format. RBApy has been used to reconstruct RBA models for wild-type and engineered *E. coli*, which demonstrate comparable growth rates on several carbon substrates [59]. This tool is available through GitHub at https://github.com/SysBioInra/RBApy, accessed on 24 June 2024. In addition, the RBAtools python package has been developed more specifically for non-modeling experts to approach RBA modeling [60]. RBAtools was used to evaluate metabolic trade-offs in *B. subtilis*, namely protein to ATP, growth to vitamin production, and fitness to ribosome concentration [60]. This tool is available through GitHub at https://sysbioinra.github.io/rbatools/, accessed on 24 June 2024. RBA models are encoded either using GAMS [16] or MATLAB [56]. RBA models have been applied to design *B. subtilis* for identifying metabolic bottlenecks and the design of de novo amino acid synthesis pathways [55], modeling seasonal variation of phytoplankton communities [61], and recapitulating protein allocation in *B. subtilis* [56], and identifying the mechanistic underpinnings of the Crabtree effect [16].

RBA models are useful in that they are the simplest of fgRAM model frameworks (though this observation does not mean that its reconstruction is simple). Rather, they are the simplest framework with an explicit metabolic cost for protein synthesis. RBA models also have automated reconstruction tools and well-described methods. Therefore, this category may be the most approachable fgRAM type for non-model organisms. Although they make simplifying assumptions, such as only explicitly synthesizing proteins and RNA, they have still proven useful to address a variety of research questions. Unfortunately, RBA models have, at present, a relatively limited repertoire of analysis tools because of their iterative LP solution methods, generally only being analyzed with resource balance analysis to date. Therefore, a key future development for this framework will be the development and application of analysis tools analogous to those in SMMs.

#### 4.2.2. Model of Metabolism and Macromolecular Expression (ME-Models)

A more detailed accounting of all macromolecules is found in the genome-scale model of metabolism and expression (ME-model). The first modeling framework for addressing macromolecular cost was the ME-model (where “ME” stands for metabolism and macromolecular expression) of *E. coli* in 2012, used to investigate codon usage bias and its relation to growth rate [62]. This modeling framework combines the metabolic network of an SMM with the synthesis of all major molecular machinery in a cell, including enzymes, mRNA, tRNA, ribosomes, cell wall, and DNA. In the first ME-model, Equation (14) is applied to all macromolecules including enzymes, tRNAs, mRNAs, RNAPs, and ribosomes, though $k_{deg,m}=0$ for all macromolecules except mRNA. In subsequent models, degradation of macromolecules used various forms, including a first-order constant (as in the case of mRNA in the first ME-model), or more complex descriptions. Further, macromolecules are not consumed, so $v_{con}=0$ for all macromolecules in ME-models.

ME-models effectively share Constraint (18) with RBA models. ME-models assume that the rate of RNA polymerase elongation ($k_{rnap}$) is three times that of peptide elongation by ribosomes (resulting from three nucleotides per peptide in codons). This assumption results in a very similar constraint for RNA synthesis, as shown below:

(19) $$k_{ribo}v_{ribo}\leq \mu \sum_{r\in R}N_{r}^{nuc}v_{r}$$

where R is the set of RNAs, $N_{r}^{nuc}$ is the length of r, and $v_{r}$ is the transcription flux of r. RNA synthesis is distributed by measured fractions of RNA consisting of rRNA, tRNA, and mRNA. ME-models also model tRNA charging, and it is assumed equal to the rate of translation in the cell (since the cell is assumed steady-state). Remaining macromolecular synthesis machinery is assumed to have a common $k_{cat}$ with synthesis rate defined by the following constraint:

(20) $$k_{cat}v_{mach}\leq \mu \sum_{m\in M^{m}}v_{m}$$

where $v_{mach}$ is the flux through the machinery synthesis equation, and $M^{m}$ is the set of macromolecular machinery where its synthesis is not already limited. In ME-models, biomass is modeled as a set of demand reactions, including for DNA, cell wall, glycogen, enzymes, RNAs, and peptides, with synthesis driven and limited by Constraint (20).

Given the complete macromolecular synthesis included in ME-modeling, ME models can be used to model synthesis costs associated with many effectors with many biological processes. Expanded ME-frameworks have been used to model stress response [63]. FoldME is an expansion of the ME-modeling framework which models protein folding and unfolding [64]. This expansion allows for modeling the effect of temperature stress on enzyme kinetics and the system-level protein reallocation. OxidizeME is another expansion of ME-models which adds the ability to model reactive oxygen species (ROS) stresses. ROS stresses are modeled through auxotrophy, damage of iron-sulfur clusters, DNA damage, and protein damage [65]. AcidifyME models pH stress through changing lipid fatty acid composition as well as protein stability and activity [23]. The new StressME framework then brings together all three expansions (FoldME, OxidizeMe, and AcidifyME) into a single stress-response model [63]. To date, the StressME framework has only been applied to *E. coli* but can be adapted with some effort to other species. Aside from being used to model stress conditions, ME-models have been used to investigate codon optimization in *E. coli* [62] and recapitulation of transcription and translation rates in *E. coli* [66].

ME-models are either solved as an iterative LP [66] or as a single-step NLP [65]. The objective function used in analysis differs. In most cases, growth maximization is the primary objective [66] (or only objective in NLP cases [65]. Where iterative LP is used to solve ME-models, the objective for each iteration may be to minimize ribosome dilution [66] or maximize synthesis of a “dummy complex” [65]. The results of these analyses are a single metabolic state, akin to flux balance analysis. In many cases, ME-models are analyzed using flux variability analysis (FVA) at a fixed growth rate [66,67,68].

Manual ME-model reconstructions are possible by following the detailed reconstruction descriptions provided in ME-modeling works [62,65,66]. A Python-based tool called COBRAme [66] is built upon the COBRApy platform for SMMs, which provides tools to simplify ME-model reconstruction and analysis. Published with COBRAme is a workflow describing ME-model reconstruction using this tool [67]. However, COBRAme is only a partially automated reconstruction tool, requiring either the ECOLIme package (an *E. coli*-specific python package containing ribosome composition and transcriptional unit definitions) or requiring equivalent manually curated inputs. COBRAme and ECOLIme packages are available through GitHub (https://github.com/SBRG, accessed on 24 June 2024). The wide breadth of the ME-Modeling framework necessitates a wide variety of data for parameterization. Proteomics, transcriptomics, catalytic turnover rates for various enzymes, and biomass distribution data are all necessary to make an accurate model. Compared to other model types discussed (see Figure 1c), the data availability is relatively low leading to unavoidable assumptions about parameter values and the use of randomization for creating synthetic data. As a result of these challenges, ME-models are few in number, and have only been reconstructed for *E. coli* [14] and *Thermotoga martima* [65,66].

ME-model reconstruction and analysis are challenging as a result of the depth of its data needs and computational complexity. However, this complication is balanced by the detail of the model and its ability to model phenomena which are not strictly metabolic, such as stresses through stressME. ME-models are, at present, the most well-developed RAM framework for studying stress and its effect on metabolism. Future research directions for ME-modeling might include frameworks for modeling signal transduction and its effect on metabolism. A particularly interesting application could be in biofilms and quorum sensing, or identifying pathways in microbial communities that are activated by signals from other community members.

#### 4.2.3. Expression and Thermodynamics Flux (ETFL) Framework

The ETFL framework was introduced in 2020 [21] as a simultaneous restructuring of the ME-Model to change from an iterative LP to a mixed-integer linear problem (MILP), as well as to integrate thermodynamic and gene expression constraints into a single modeling framework. Similar to the ME-model framework [20], major cell machinery components are explicitly modeled, with the only notable absentee being the cell wall. As with RBA models, a “dummy” protein is used to account for the metabolic cost of unmodeled proteins [21]. The thermodynamic constraints come from the thermodynamics-based metabolic flux analysis (TMFA) framework [69], also an MILP tool for SMMs. TMFA is defined as follows:

$$constraints\;\left(1\right)\;to\;(3)$$

(21) $$\Delta_{r}G_{j}^{\prime }=\Delta_{r}G_{j}^{\prime o}+RT\sum_{i\in I^{r}}S_{ij}C_{i}\;\;\;\;\;\;\;\;\;\;\;\;\;\;\;\;\;\;\;\forall j\in J$$

(22) $$\Delta_{r}G_{j}^{\prime }-M+Mb_{j}^{+}\leq 0\;\;\;\;\;\;\;\;\;\;\;\;\;\;\;\;\;\;\;\;\;\;\;\;\;\;\;\;\;\;\;\;\;\forall j\in J$$

(23) $$-\Delta_{r}G_{j}^{\prime }-M+Mb_{j}^{-}\leq 0\;\;\;\;\;\;\;\;\;\;\;\;\;\;\;\;\;\;\;\;\;\;\;\;\;\;\;\;\;\forall j\in J$$

(24) $$v_{j}^{+}-Mb_{j}^{+}\leq 0\;\;\;\;\;\;\;\;\;\;\;\;\;\;\;\;\;\;\;\;\;\;\;\;\;\;\;\;\;\;\;\;\;\;\;\;\;\;\;\;\;\;\;\;\;\forall j\in J$$

(25) $$v_{j}^{-}-Mb_{j}^{-}\leq 0\;\;\;\;\;\;\;\;\;\;\;\;\;\;\;\;\;\;\;\;\;\;\;\;\;\;\;\;\;\;\;\;\;\;\;\;\;\;\;\;\;\;\;\;\;\forall j\in J$$

(26) $$b_{j}^{+}+b_{j}^{-}\leq 1\;\;\;\;\;\;\;\;\;\;\;\;\;\;\;\;\;\;\;\;\;\;\;\;\;\;\;\;\;\;\;\;\;\;\;\;\;\;\;\;\;\;\;\;\;\;\;\;\;\;\forall j\in J$$

where $\Delta_{r}G_{j}^{\prime o}$ is the standard Gibbs free energy of reaction (generally estimated by the group contribution method [70]), R is the ideal gas constant, T is the temperature in Kelvin, $C_{i}$ is the log-fold difference in concentration of metabolite i from some reference state, M is an arbitrary large number, $b_{j}^{+}$ is a binary variable with a value of 1 if the reaction can proceed in the forward direction, $b_{j}^{-}$ is a binary variable with a value of 1 if the reaction can proceed in the reverse direction (mutually exclusive with $b_{j}^{+}$). Constraint (21) defines the Gibbs energy of the reaction. Constraints (22) and (23) ensure the sign of the Gibbs energy of the reaction matches with the direction that the reaction proceeds in. Constraints (24) and (25) block or allow a certain direction that the reaction flux can proceed in. Constraint (26) ensures that the reaction will only progress in one direction.

A primary assumption made within the ETFL model that is notably absent from the ME-model formulation is that the dilution rate of metabolites and the degradation rate of certain “stable” macromolecules are negligible. The latter manifests in Constraint (14), in that $k_{deg,m}=0$ is assumed for all macromolecules. These assumptions reduce the need to linearize certain portions of the problem. A novel addition to the framework is the normalization of all variables to an assumed maximum value, as the ranges for synthesis reaction fluxes of the modeled components (from 10^−10^ to 10^1^) can cause optimal solutions to occur outside of a solver’s accuracy limit (typically 10^−9^).

Linearizing the general formulation is done by performing discretization on the growth rate in such a way that allows the conversion of the dilution term for macromolecules into a linear term with integer-based constraints. The term $\mu e_{m}$ in Equation (14) represents the concentration loss resulting from dilution, in order to linearize this term, the growth rate is discretized, as shown in Constraints (27) and (28) below:

(27) $$\mu =\frac{\;p\hat{\mu }}{N},$$

(28) $$p=\Sigma_{s=0}^{\left\lceil \log_{2}N\right\rceil }2^{s}\delta_{s}$$

where N represents the desired number of discrete levels for the growth rate, and the parameter $\hat{\mu }$ is an estimated maximum growth rate. The value of p, based on the values of binary variables $\delta_{s}$, decides which discrete value for the growth rate (between 0 and the maximum growth rate) is chosen. In doing so, the Petersen linearization scheme can be used to convert the dilution term into a single variable $z_{*}^{s}$ with linear constraints based on $e_{j}$ and $\delta_{s}$ variables, as seen in Equations (29)–(31). A similar formulation can be used for other growth dependent bilinearities, such as the growth-rate-to-protein ratio.

(29) $$e_{j}+M\delta_{s}-z_{*}^{s}\;\leq M$$

(30) $$\;\;\;\;\;z_{*}^{s}-M\delta_{s}\;\leq 0$$

(31) $$z_{*}^{s}-e_{j}\leq 0$$

Models using the ETFL framework are solved via MILP, in all cases assuming maximization of growth rate of wild-type strains, which results in a single metabolic state [21,71]. ETFL models have been analyzed using FVA and an adaptation of the minimization of metabolic adjustment (MOMA) called the minimization of protein adjustment (MOPA), which assumes that knockout strains will show a minimal change in protein distribution compared to the wild-type [21].

Not only does the ETFL framework have the data requirements of the ME-Modeling framework, but the introduction of thermodynamic constraints also requires metabolite concentration data. Although not strictly necessary, as the data is used to place limits on metabolite concentration and can be roughly estimated, the accuracy of the model heavily depends on these limits, and thus would benefit from estimates curated from metabolomic data. This component gives the ETFL framework the largest data requirement of any model discussed (see Figure 1c). Because of its complexity, high data requirements, and corresponding lack of automated reconstruction tools, to date, ETFL models have been reconstructed only for two species: *E. coli* and *S. cerevisiae* [71]. To date, ETFL models have generally been limited in use to recapitulating growth rate, gene essentiality and overflow metabolism phenotype [21,71].

Models using the ETFL framework are the most complex RAMs discussed here to reconstruct because of its need for not only metabolic, fluxomic, and proteomics datasets, but also data for characterizing in vivo thermodynamics. This burden, along with high computational complexity, are the trade-offs of a highly detailed and informative model. Whereas SMMs and other fgRAM models are good at recapitulating or estimating product yield, in some cases product titer (dependent on thermodynamics) can be a considerable limitation to the economic viability of bioproduction platforms such as *Clostridium thermocellum* for ethanol [72]. With the incorporation of concentration and thermodynamics, ETFL models have the best potential to address limitations of product titer. However, given the high complexity and data needs of this framework, ETFL models may be slow to be adopted unless programming packages, semi-automated reconstruction workflows, protocols, and methods of estimating missing data are developed to speed model reconstruction and breadth of application and build a community of expertise.

## 5. Discussion and Conclusions

We discussed several SMM tools and RAM frameworks for connecting proteome distribution and/or macromolecule synthesis cost to metabolism. The contents of these various frameworks are summarized in Figure 3 and their respective constraints are contrasted in Table 1. These efforts began simply with the addition of cellular volume limitation constraints within FBA termed molecular crowding (FBAwMC) applied to SMMs. Since then, models have expanded to include additional factors including the synthesis and dilution of all major macromolecules including enzymes (e.g., RBA framework), RNA, DNA, lipids, and ribosomes (e.g., ME-model), and models which incorporate thermodynamics (e.g., ETFL framework).

Each model reconstruction framework has strengths and weaknesses, though generally there exists a trade-off between model complexity, computational complexity, and data needs (where each increases with the others) which prevents all but model organisms from being reconstructed using the most detailed frameworks. In general, cgRAM models are easier to reconstruct with more available protocols and tools to further ease reconstruction. cgRAM models are limited in that they do not impose a direct metabolic burden for protein synthesis and will be most useful to answer questions or test hypotheses related to protein distribution. On the other hand, fgRAM models are more complex. RBA models are the simplest category of these, which impose metabolic cost of proteins, RNA, and ribosomes to limit metabolism. Other fgRAM frameworks, such as ME and ETFL, either have or have great potential for specialization for linking metabolism to phenotypes which other modeling frameworks cannot address. ME-models for instance can model oxidative (oxidizeME), acidic (acidifyME), and protein unfolding stress (foldME). On the other hand, ETFL models include reaction thermodynamics, which is not considered by other models and has the potential to address issues of product titer limitations. A mathematical comparison of model frameworks, highlighting their different constraints and the roles of those constraints, is provided in Table 1.

As happened before with SMMs, RAMs are following a similar pattern of first being reconstructed for species with abundant data and well-understood metabolism, such as *E. coli*, *B. subtilis*, and *S. cerevisiae*. Again, similar to SMMs, this early work has been followed by automated reconstruction tools and diversified into other organisms. Unlike SMMs, RAM frameworks can in principle model the complete flow of information through the central dogma of biology. Doing so requires accounting for the synthesis of macromolecular machinery (ribosomes, tRNA, etc.) and processes such as protein degradation, protein folding, and oxidative stress. As noted in Figure 1, these inclusions could enable addressing several new research questions. In analogy to how SMMs tended to call different tools for different tasks. For some model types, like ME-models and ETFL models, this process has already begun with adaptations of flux variability analysis and MOMA. Future tool development will likely include analogs to OptKnock (a tool which uses gene knockouts and reaction eliminations to design strains with growth-linked production) [73], OptStoic (a tool for identifying and designing pathways for production) [74], and OptCom (for microbial community simulation) [75].

Of the two classes of resource allocation models, fgRAM models are much less standardized in format usually relying on MATLAB, Python, and GAMS implementations. Some models are stored as text files [16] whereas others [48] are available in SBML format. We believe a key development moving forward will be the standardization of fgRAM models. In the same way that SMMs took many early forms, now are all largely formatted in SBML. Such standardization would have several key advantages including interoperability, ease of use on different platforms (for instance, MATLAB and Python COBRA packages both read SBML files), and ease of using a simpler RAM as the basis for reconstruction of a more complex type.

Another key challenge in this area is the estimation of apparent kinetic $k_{app}$ parameters. These are concentration-dependent and not the same as $k_{cat}$ for which typically ML tools can provide estimates [76]. Considering the Michaelis-Menten equation, shown below, $k_{app}$ will generally be less than $k_{cat}$ by the extent of substrate saturation.

(32) $$v_{j}=\frac{k_{cat}e_{j}c_{j}}{K_{M}+c_{j}}=k_{app}e_{j}$$

(33) $$k_{cat}\left(\frac{c_{j}}{K_{M}+c_{j}}\right)=k_{app}$$

Therefore, $k_{app}$ is not only dependent on the enzyme, but the state of its environment (metabolite saturation in parentheses in Equation (33)). Thus, either a workflow needs to be developed to estimate apparent kinetic parameters, making assumptions of enzyme and metabolite distribution as done in [16] or $k_{cat}$ values are accepted as surrogates for $k_{app}$ with the acknowledgement that it will be an over-estimate of kinetics. What complicates the picture further is that studies have shown evidence for the presence and operation of metabolons that enhance locally metabolite concentrations [16] boosting $k_{app}$ values even above in vitro derived $k_{cat}$ values. On the parameterization end, a key concern is the use of mean or median $k_{cat}$ or $k_{app}$ values when these values are unknown as is done in AutoPACMEN and in RBA model reconstruction workflows [16,44]. Sensitivity of the obtained results on the adopted mean values should aways be carried out as a cautionary check. As mentioned before $k_{cat}$ values are measured under in vitro conditions. However, it has been shown before that the correlation between in vitro and in vivo activity is generally weak. Studies have inferred for *S. cerevisiae* a correlation value of $R^{2}=0.28$ [77], for *E. coli* $R^{2}=0.62$ [78] and for *Arabidopsis thaliana* $R^{2}=0.45$ (compared to median BRENDA $k_{cat}$) [79]. Therefore, directly inferring $k_{app}$ values using quantitative in tandem fluxomic and proteomic data $v=k_{app}\;[E]$ should be the golden standard. This gold standard has several advantages, including bypassing the issue inconsistent kinetics cause by of intrinsically disordered-domains, since it results in net apparent kinetics of the in vivo system, compared to ideal kinetics measured in vitro. Nonetheless, this process is still an estimate of kinetics, which may change based on metabolic state, and effective kinetics may be different under different growth conditions and the linear approximation would likely fail far from the state at which $k_{app}$ is estimated. Further, $k_{app}$ estimates may be influenced by noise and heterogeneity in both protein abundance and reaction rate (^13^C MFA) measurements. Despite the potential for inaccurate $k_{app}$ estimates, few RAM model investigations include parameter sensitivity analyses. Model sensitivity to kinetics has been investigated in multiple ways, including substituting known $k_{app}$ values for average values [21] and perturbing the effective kinetic parameter by an order of magnitude in either direction [65]. A robust analysis of a RAM model should use this gold standard as a baseline, then apply sensitivity analysis to determine if model conclusions are valid under different kinetic estimates.

## 6. Future Directions

A key area of investigation currently lacking in RAM frameworks applications is investigation of multicellular organisms and microbial communities. This scope is understandable as RAM modeling is following a similar trajectory to the early years of SMMs. In part this limitation arises from the lack of abundance of data, (relative) simplicity of the modeled organism, and (relative) ease of hypothesis testing. However, we foresee key research questions addressable by RAM models in eukaryotic and multicellular organisms with great potential to impact diverse fields, particularly medicine, microbial cellular factories, agriculture, or even questions around the evolution of life.

In medicine, metabolism in cancer is a ripe area of investigation using RAM frameworks. It has long been noted that cancer cells exhibit unique metabolic phenotypes, such as the Warburg effect [80], and many have argued that cancer is a metabolic disease [48]. With significant metabolic reprogramming in tumor cells during disease progression [81], large-scale proteomic changes (for instance, a shift toward anaerobic respiration proteins such as lactate dehydrogenase) could be used to identify new therapeutic targets unique to cancer cells. Sequences from cancer cell biopsies along with tools such as SNPeffect (which uses metabolic models to identify functional roles of SNPs) [82] can be used to create tumor-specific metabolic networks. In conjunction with drug-target interaction databases, these networks can be screened for metabolic protein targets which are uniquely susceptible in the cancer cell.

In cellular factories producing bioproducts or biofuels, whereas SMMs are adept at determining yield, they are unable to infer titer or capture product feedback inhibition [83,84]. Therefore, although an SMM-designed strain may have high yield under low product titer, it could fail to have economically viable titer by the end of a fermentation process. Here, ETFL models, which already incorporate thermodynamics, can be used to identify which pathway steps become highly reversible or irreversible at high product titer, and in what order. The steps most susceptible to high product titer can be replaced with steps from other organisms which have higher thermodynamic driving forces. The model can be used to drive investigation into rebalancing metabolism in cases where substitutions result in new cofactor stresses.

In agricultural applications, heat-stressed plant enzymes suffer higher protein turnover (from degradation and damage) [37], increased antioxidant enzyme activity [85], and change in enzyme activity (including RUBISCO [86]). Each of these imposes protein-associated metabolic burdens [87], or is a protein-associated metabolic bottleneck [86] which require RAM-centric descriptions. The foldME and stressME tools are ideally suited for these investigations. A particularly interesting research question here could include to what extent the sub-optimal phenotype of heat-stressed plants results from the energetic cost of protein refolding compared to the increased oxidative stress associated with both heat and drought. The study could highlight the costliest enzymes as reengineering targets and hypothesize phenotype improvements from more stable enzymes.

In investigating the origins of life problem, it is hypothesized that early life utilized catalytic RNA machinery to drive its metabolism, and possibly for storing genetic information [88]. RAM models of hypothetical early lifeforms could be reconstructed (perhaps using a global “dummy” sequence at first, similar to what is suggested for improving GECKO and MOMENT frameworks). These models could investigate the cost of metabolic catalysis in early life and be used to model efficiencies from the transition to protein-based machinery. Several interesting research questions arise here including: (i) how much more “fit” would a protein-using organism be than an RNA using one? (ii) how much metabolic energy in early lifeforms would have to be dedicated to maintaining its RNA machinery (e.g., re-folding or replacing degraded molecules)? (iii) how would the acidic, reducing environment of Earth encountered by early life forms stress such an organism?

Another key opportunity is the development of integrated kinetic-resource frameworks. To elaborate, RAM models provide the most basic linear kinetic approximation as a bound to reaction rates while simultaneously estimating the rate of synthesis needed to maintain steady state. If it is assumed that there is neither enzyme degradation nor consumption ($k_{deg,m}=0$ and $v_{conc}$ is assumed negligible in Constraint (14)) then this synthesis rate is proportional to enzyme concentration. Allowing variable enzyme synthesis would then allow for an estimation of enzyme concentration from growth rate, which is fixed at each iteration if using RBA or ME-model frameworks. An iterative framework between kinetic models (determining metabolite concentration and determining $k_{app}$) and resource allocation models (for determining enzyme concentration) could be used to create a highly parameterized model of a target organism which integrates the two model types.

Finally, to this point, all models discussed are metabolic “snapshots” at pseudo-steady state, giving a single metabolic state or evaluating the breadth of feasible metabolic states. Further, models account for the metabolic cost of transcription and translation, but not its regulation. However, by modeling the flow of information in a cell, fgRAMs, particularly ME and ETFL models, can model transcriptional and translational regulatory networks, as has been suggested [71,89], but not yet implemented. Although it would no doubt be computationally costly, dynamic fgRAM models could incorporate gene regulatory network models to capture metabolic dynamics in transitional state. This area holds the promise of designing inducible metabolic systems.

## Figures and Tables

**Table 1:** Constraints involved with resource analysis frameworks to compare and contrast what is modeled by different frameworks discussed here. Yellow cells indicate the constraint present in the SMM tool or RAM framework. This table also notes the type of problem for each tool or framework.

Framework Category							Constraints	
Precursor		cgRAM		fgRAM				
FBAwMC	FBAwSCC	GECKO	MOMENT	RBA	ME Model	ETFL	Conceptual Description	Eqn. No.
×	×	×	×	×	×	×	Objective function	(1)
×	×	×	×	×	×	×	Mass balance	(2)
×	×	×	×	×	×	×	Flux bounds	(3)
×							Molecular crowding	(4)
	×						Solute capacity	(5)
		×	×	×	×	×	Linear enzyme kinetics limitation	(6)
		×	×	×	×	×	Enzyme capacity	(9)
			×				Enzyme pool determination	(10)
			×				Enzyme pool limit	(11)
				×	×	×	Macromolecule mass balance (pseudosteady-state)	(14)
				×	×		rRNA capacity constraint	(17)
					×		Protein-ribosome coupling constraint	(18)
				×			Transcription capacity constraint	(19)
					×		Macromolecular machinery capacity constraint	(20)
						×	Thermodynamic constraints on reaction direction	(21)–(26)
						×	Petersen linearization of growth-driven dilution	(29)–(31)
LP	LP	LP	LP	Iterative LP	Iterative LP or NLP	MILP	Type of problem	

## Data Availability

Not applicable.
